# Risk Factors of the Invasive Breast Cancer Locoregional Recurrence

**DOI:** 10.1155/2015/789646

**Published:** 2015-08-03

**Authors:** R. V. Liubota, A. S. Zotov, R. I. Vereshchako, I. I. Liubota, V. V. Zaychuk

**Affiliations:** ^1^Department of Oncology, National Medical University, 13 Boulevard Shevchenko, Kiev 01030, Ukraine; ^2^Kiev Oncology Municipal Centre, Kiev, Ukraine

## Abstract

*Background*. The aim of the research was to estimate the frequency of the locoregional breast cancer recurrence appearance, the recurrence-free period continuance, and the 3- and 5-year survival depending on the scope of the surgical intervention, menstrual profile, and histological and molecular-biologic characteristics of the primary tumor. *Patients and Methods*. Among 218 patients with a breast cancer, 99 patients had breast-conserving surgery (BCS) and 119 underwent radical mastectomy (RME); all patients had regional lymphatic nodes dissection. The size and the primary tumor differentiation degree, metastasis presence in the regional lymph nodes, ER expression, PR, and Her/2neu were assessed as the prognostics factors. *Results*. It was defined that the locoregional recurrence appearance frequency in patients with BCS turned out to be 13%, and in patients after RME it turned out to be 9%; the recurrence-free period continuance was 53 ± 8 months and 56 ± 10 months, respectively. *Conclusions*. The locoregional cancer recurrence frequency is higher in women with the menstrual function being preserved at the moment of the primary tumor detection than in postmenopausal patients and also in patients having the hyperexpression of the Her/2neu. The ipsilateral cancer recurrence decreases the 3-year survival by 7,1% and the 5-year one by 20,3%, respectively.

## 1. Introduction

According to the GLOBOCAN 2012 v1.0, in 2012, worldwide there were 14.1 million new cancer cases, 8.2 million cancer deaths, and 32.6 million people living with cancer (within 5 years of diagnosis). 57% (8 million) of new cancer cases, 65% (5.3 million) of the cancer deaths, and 48% (15.6 million) of the 5-year prevalent cancer cases occurred in the less developed regions. Breast cancer is the second most common cancer in the world and, by far, the most frequent cancer among women with an estimated 1.67 million new cancer cases diagnosed in 2012 (25% of all cancers), where it is the most frequent cause of cancer death in women in less developed regions (324,000 deaths, 14.3% of total), and it is the second cause of cancer death in more developed regions (198,000 deaths, 15.4%) after lung cancer [1].

The successes in diagnostics and chemoradiotherapy of the breast cancer at the present stage of the oncology development lead to the reconsideration of the surgical treatment methods. At first, Maddox et al. [2] showed that the overall survival (OS) and disease-free survival (DFS) indices do not essentially differ among the patients who passed the radical mastectomy (RME) on Halsted and the modified mastectomy on Patey. In 1970-80, after comprehensive analysis, few clinical trials results in breast surgery prevailed the tendency to the elaboration of the breast-conserving surgery [3–9]. At the present time, to cure the BC, the modified radical mastectomies (Patey and Madden) or the breast-conserving surgery (lumpectomy and quadrantectomy) are often used. While choosing between RME and breast-conserving surgery (BCS), the main problem, both to the doctor and to the patient, is to reach the maximum cosmetic result at the minimum local recurrence risk. This is possible only with presence of the tumor occupying up to 25% of the breast size and upon the condition that the “clean” margin of excision is reached. The local recurrences after BCS require the subsequent surgical treatment (more frequently, mastectomy) neutralizing the reached cosmetic result, and being the indices of the tumor aggression and a high degree of the distant metastasis availability [10–12]. For this reason, when treating the breast cancer, the determination of the recurrence risk factors should directly influence the surgical interference volume choosing process.

## 2. Patients and Methods

218 patients had been examined at the age of 31 to 92 (57 ± 1, 3) years, who passed BC treatment course at the oncological clinic of the National Medical University in Kiev Oncology Municipal Hospital in 2004–2009. Among them, 162 patients were treated from primary BC in 2004-2005, and 56 patients were treated from BC recurrence in 2004–2009. The patients were divided into 2 groups: to the first group (*n* = 99) the patients who underwent the BCS were referred (lumpectomy: 35 and quadrantectomy: 64, both with the regional lymphatic nodes dissection), and to the second group (*n* = 119) we referred the patients who passed RME (RME by Madden: 91 and RME by Patey-Dyson: 28). In their turn, groups 1 and 2 were divided into 2 subgroups. To group A were referred the patients without breast cancer recurrence and to group B the patients with the recurrence appearance. Patients were assigned to the BCS with an attempted margin of 1 cm of healthy tissue. Margins were routinely inked to assess the microscopic completeness of the lumpectomy. The criteria to choose for each patient BCS or RME are tumor size, extensive DCIS, tumor margins, tumor location, need for radiation, risk reduction, and individual needs and preferences. Reasons to avoid BCT include multiple tumors, extensive tumor, and contraindication for radiation.

To identify the 3- and 5-year survival, the patients were divided into the following groups: the main group consisting of 154 women who did not have the locoregional breast cancer recurrence during the supervision period and the second group consisting of patients who experienced the locoregional breast cancer recurrence. The same groups were used in the assessment of the menstrual function influence on the recurrence frequency.

The cuts of 4-5 *μ*m in thickness were made of paraffin bricks (standard procedure of haematoxylin-eosin preparation) and placed on the glasses treated with poly-L-lysine. Then, the material was treated according to the standard procedure using the following antibodies: ER-clone 1D5, PgR-clone 636, and Her-2/neu-clone Cb11. The results interpretation was done by immunohistochemical reaction using the nuclear reaction qualitative assessment: the negative “–,” the low positive “+,” moderately positive “++,” strongly positive “+++,” and quantity of dyed tumor cells in %.

In making an assessment of Her-2/neu expression, the intensity of cytoplasmic basal membrane coloring was pointed out: the reactions “−” and “+,” the absence of hyperexpression, and the reaction “+++,” hyperexpression of Her-2/neu. The presence of the hyperexpression Her-2/neu in cases of “++” reaction is conducted with the help of hybridization method in situ using the fluorescent marker FISH (fluorescent in situ hybridization). The investigations were conducted at the pathohistological laboratory at Kiev Municipal Oncology Hospital.

The patients received adjuvant systemic treatment and radiotherapy according of the St. Gallen International Breast Cancer Conference recommendations (2001–2005).

The histopathological diagnosis of breast recurrences was reviewed and compared with that of the initial tumor taking into account cytological, morphological/architectural, and stromal patterns, histological grade, and immunohistochemical staining (hormonal receptors, c-ErbB2). The majority of these parameters had to be similar for a given lesion to be declared a true recurrence.

## 3. Statistical Methods

In estimating the influence of the axillary node involvement, histological grade, tumor size, and immunohistochemical staining (hormonal receptors and c-ErbB2), the fourfold tables analysis method and the 2 × *K* tables analysis were used.

The connection between the LR BC and the menstrual function condition of the patients at the primary tumor detection was defined via 2 × 2 tables' analysis.

The Kaplan-Mayer method was used to estimate the patient's survival rate [13].

## 4. Results

**Figure 1:**
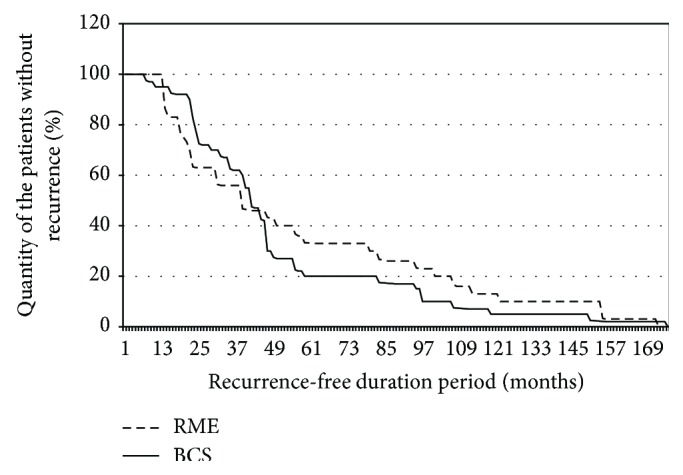
Recurrence-free duration period.

Table 1 shows clinicopathological information for the 218 enrolled patients. It was established that the frequency of the local recurrence appearance after BCS and RME (1 group) turned out to be 13%, and in patients after radical mastectomy (2 group) it turned out to be 9%. The recurrences frequency in this research corresponds to that of leading oncology hospitals: thus, according to the data from 6 prospective randomized researches [9–12, 14, 15], the recurrences frequency after the mastectomy ranges from 4 to 18%. The recurrence-free period duration in group 1 turned out to be at an average of 53 ± 8 months in the breast-conserving surgery group and 56 ± 10 months in the RME group. The minimum period of BC recurrence appearance after the BCS was 9 months (after RME, 10 months), and the maximum period was 177 months in group 1 against 174 months in the second one (Figure 1).

The tumor size, axillary node involvement, and histological grade are the predictive factors of the disease run [14]. In the Tables 2, 3, and 4, the distribution of patients according to the tumor size, axillary node involvement, and histological grade, respectively, is shown. It was established that the tumor size, axillary node involvement after mastectomy, and histological grade do not influence the frequency of the BC recurrence at the level of significance (*p*) 0,05. But in the BCS group the axillary node involvement increases the frequency of the ipsilateral recurrence of the breast cancer.

The results of the immunohistochemical examination are shown in the Table 5. Evaluating the tumor receptor status used the 2 × *K* table analysis method [13]. It was established that the primary tumor receptor status (ER, PR) in groups 1 and 2 and the expression degree Her/2neu after the RME do not influence the BC recurrence. In breast-conserving surgery group, the hyperexpression of Her/2neu increases the frequency of the locoregional breast cancer recurrence appearance.

**Figure 2:**
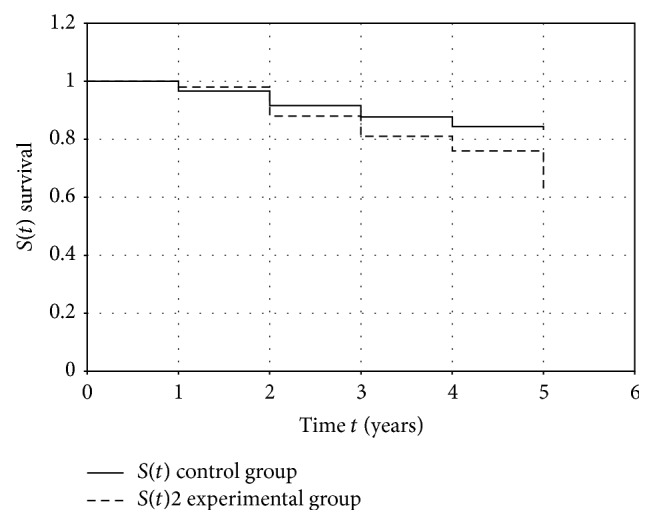
Patient survival curves according to the BC recurrence presence or absence.

The assessment procedure of Kaplan-Meyer was used to evaluate the overall patients' survival. Three-year survival rate in patients without BC recurrence was 87,6% and the 5-year survival rate was 82,8%. Three- and 5-year survival in patients with BC recurrence of the BC corresponded to 80,5% and 62,5%, respectively (Figure 2). The differences in 3-year survival between groups are not statistically significant (*p* > 0,05) and differences in 5-year survival between groups are statistically significant (*p* < 0,05).

## 5. Discussion

The era of conserving surgeries in breast cancer started in the 1970s. U. Veronesi in Milan Cancer Institute (Italy) and B. Fisher in The Abramson Cancer Center of the University of Pennsylvania (USA) proposed independently of each other to perform the conserving surgery in breast cancer. In 1969, results of randomized studies to compare radical mastectomy with breast-conserving surgery, which was termed “quadrantectomy,” were approved by the World Health Organization Committee of Investigators for Evaluation of Methods of Diagnosis and Treatment of Breast Cancer [15]. The recruitment of patients began at the Milan Cancer Institute in 1973, after the new procedure was standardized, and preliminary data showing that survival rates were equal after radical and breast-conserving surgery were published in 1977 and 1981 [5, 7]. In 1971, the National Surgical Adjuvant Breast and Bowel Project (NSABP) initiated the B-04 study, a randomized clinical trial conducted to resolve controversy over the surgical management of breast cancer.

The afterward published results of the above investigations had not demonstrated the appreciable difference of the late fates. However, in patients after the BCS, the probability of the locoregional recurrence appearance is higher compared with the patients who have been undergone the radical mastectomy. The recurrence appearance requires another surgical intervention, oftener than the mastectomy which aligns the cosmetic an d esthetic effect reached at BCS. Besides, the appearance of the regional breast cancer decreases the 3- and 5-year survival.

The important thing in the clinical practice is the drawing distinctions between the real recurrences and the newly occurred ipsilateral tumors. This is due to the fact that the newly occurring breast cancer is susceptible to the X-ray therapy and standard schemes of the chemotherapy, whereas the recurrent tumor is chemo- and radio-resistant and requires the adjuvant treatment modification [9, 14, 15].

The causes of the ipsilateral recurrences development could be the following ones: tumor cells expansion along the muscle fiber, fascial plates, vessels, and nerve and perineural crevice tunics; the tumor could have plural rudiments (multicentricity and multifocality), which are the causes of underestimation of the process expansion; it could be rested in the edges the microscopic normal, but genetically changed cells, which will initiate the recurrent tumor development.

The presence of the established risk factors of breast cancer recurrence development in patients (the presence of metastases in the regional lymph nodes and the hyperexpression of Her/2neu in the primary tumor cells) requires more strict control of the adjuvant treatment; the main efforts must be directed to the early recurrence detection and the usage of the up-to-date methods of diagnostics (MRT, PET) and biopsy at the susceptible recurrent focuses.

## 6. Conclusion

1. The surgical intervention volume does not affect the frequency of the locoregional breast cancer recurrence appearance.
2. The recurrence-free period duration in patients who have been done the breast-conserving surgery does not essentially differ from the patients who have been undergone the mastectomy.
3. In patients after the breast-conserving surgery, the presence of metastases in the regional lymphatic nodes and the hyperexpression Her/2neu in the primary tumor cells is associated with the higher risk of the locoregional recurrence.
4. The regional breast cancer appearance decreases the 3-year survival by 7,1% and 5-year survival by 20,3%.

## Figures and Tables

**Table 1:** Clinicopathological features for the examined patients.

Characteristic	Group 1	Group 2	*p* value
	*n* = 99	*n* = 119	
Age			
<50	42 (42%)	43 (36%)	>0,05
50–69 years	48 (48%)	70 (59%)	
≥70 years	9 (42%)	6 (5%)	
Menopausal status			
Premenopausal	67 (68%)	69 (58%)	>0,05
Postmenopausal	32 (32%)	74 (36%)	
Tumor size			
≥2 cm	73 (74%)	35 (29%)	<0,05
2,1–5 cm	26 (26%)	84 (71%)	
Tumor histological grade			
High (G1)	14 (14%)	7 (6%)	>0,05
Middle (G2)	73 (74%)	98 (82%)	
Low (G3)	10 (10%)	11 (9%)	
Undifferentiated (G4)	2 (2%)	3 (3%)	
Regional lymph nodes involved			
No	61 (62%)	69 (58%)	>0,05
Yes	38 (38%)	50 (42%)	
ER presence			
Negative	35 (35%)	46 (39%)	>0,05
Positive	64 (65%)	73 (61%)	
PR presence			
Negative	37 (37%)	51 (43%)	>0,05
Positive	62 (63%)	68 (57%)	
Her2/neu presence			
Negative	71 (72%)	99 (83%)	>0,05
Positive	28 (28%)	20 (17%)	
Breast cancer subtypes			
Luminal A	47 (48%)	73 (61%)	<0,05
Luminal B	17 (17%)	12 (10%)	>0,05
Her2-positive	11 (11%)	8 (7%)	
Triple negative	24 (24%)	26 (22%)	

**Table 2:** The primary tumor size.

Examined group	Tumor size		Total
	Till 2 cm	2–5 cm	
1 A group	56^∗^ (26%)	9^∗^ (4%)	65 (30%)
1 B group	27^∗^ (12%)	7^∗^ (3%)	34 (15%)
2 A group	23^∗^ (11%)	66^∗^ (30%)	89 (41%)
2 B group	12^∗^ (6%)	18^∗^ (8%)	30 (14%)
Total	118 (55%)	100 (45%)	218 (100%)

^∗^The differences between groups are not statistically significant (*p* > 0,05).

**Table 3:** The primary tumor histological grade.

Examined group	Tumor histological grade				Total
	High	Middle	Low	Undifferentiated	
1 A group	12^∗^ (5,5%)	44^∗^ (20%)	8^∗^ (4%)	1^∗^ (0,5%)	65 (30%)
1 B group	2^∗^ (1%)	29^∗^ (13%)	2^∗^ (1%)	1^∗^ (0,5%)	34 (15,5%)
2 A group	5^∗^ (2%)	73^∗^ (33,5%)	9^∗^ (4%)	2^∗^ (1%)	89 (40,5%)
2 B group	2^∗^ (1%)	25^∗^ (11,5%)	2^∗^ (1%)	1^∗^ (0,5%)	30 (14%)
Total	22 (9,5%)	170 (78%)	21 (10%)	5 (2,5%)	218 (100%)

^∗^The differences between groups are not statistically significant (*p* > 0,05).

**Table 4:** The presence of the metastases in the regional lymph nodes.

Examined group	Axillary node involvement			Total
	The metastases in the regional lymph nodes are not present	The metastases in the regional lymph nodes are present in 1–3 nodes	The metastases in 4 and more regional lymph nodes	
1 A group	47^∗∗^ (22%)	11^∗∗^ (5%)	7^∗∗^ (3%)	65 (30%)
1 B group	14^∗∗^ (6,5%)	12^∗∗^ (5,5%)	8^∗∗^ (4%)	34 (16%)
2 A group	55^∗^ (25%)	18^∗^ (8%)	16^∗^ (7%)	89 (40%)
2 B group	14^∗^ (6,5%)	9^∗^ (4,5%)	7^∗^ (3%)	30 (14%)
Total	130 (60%)	50 (23%)	38 (17%)	218 (100%)

^∗^The differences between groups are not statistically significant (*p* > 0,05).

^∗∗^The differences between groups are statistically significant (*p* < 0,05).

**Table 5:** Immunohistochemical staining of the primary node.

Examined group	ER		PR		Her2/neu	
	Positive	Negative	Positive	Negative	Positive	Negative
1 A group	38^∗^ (39%)	22^∗^ (22%)	43^∗^ (43%)	16^∗^ (17%)	2^∗∗^ (2%)	58^∗∗^ (59%)
1 B group	26^∗^ (26%)	13^∗^ (13%)	20^∗^ (20%)	20^∗^ (20%)	26^∗∗^ (26%)	13^∗∗^ (13%)
2 A group	55^∗^ (46%)	26^∗^ (22%)	50^∗^ (42%)	31^∗^ (26%)	13^∗^ (11%)	68^∗^ (57%)
2 B group	18^∗^ (15%)	20^∗^ (17%)	18^∗^ (15%)	20^∗^ (17%)	7^∗^ (6%)	31^∗^ (26%)

^∗^The differences between groups are not statistically significant (*p* > 0,05).

^∗∗^The differences between groups are statistically significant (*p* < 0,05).

## References

[1] Ferlay J., Soerjomataram I., Ervik M. (2013). *GLOBOCAN 2012 v1.0, Cancer Incidence and Mortality Worldwide: IARC CancerBase no. 11*.

[2] Maddox W. A., Carpenter J. T., Laws H. L. (1983). A randomized prospective trial of radical (Halsted) mastectomy versus modified radical mastectomy in 311 breast cancer patients. *Annals of Surgery*.

[3] Blichert-Toft M., Nielsen M., Düring M. (2008). Long-term results of breast conserving surgery vs. mastectomy for early stage invasive breast cancer: 20-year follow-up of the Danish randomized DBCG-82TM protocol. *Acta Oncologica*.

[4] Wapnir I. L., Dignam J. J., Fisher B. (2011). Long-term outcomes of invasive ipsilateral breast tumor recurrences after lumpectomy in NSABP B-17 and B-24 randomized clinical trials for DCIS. *Journal of the National Cancer Institute*.

[5] Veronesi U., Cascinelli N., Mariani L. (2002). Twenty-year follow-up of a randomized study comparing breast-conserving surgery with radical mastectomy for early breast cancer. *The New England Journal of Medicine*.

[6] Sarrazin D., Lê M., Rouëssé J. (1984). Conservative treatment versus mastectomy in breast cancer tumors with macroscopic diameter of 20 millimeters or less: the experience of the Institut Gustave-Roussy. *Cancer*.

[7] Fisher B., Anderson S., Bryant J. (2002). Twenty-year follow-up of a randomized trial comparing total mastectomy, lumpectomy, and lumpectomy plus irradiation for the treatment of invasive breast cancer. *The New England Journal of Medicine*.

[8] Straus K., Lichter A., Lippman M. (1992). Results of the National Cancer Institute early breast cancer trial. *Journal of the National Cancer Institute. Monographs*.

[9] Livi L., Paiar F., Saieva C. (2007). Survival and breast relapse in 3834 patients with T1-T2 breast cancer after conserving surgery and adjuvant treatment. *Radiotherapy & Oncology*.

[10] Blichert-Toft M., Rose C., Andersen J. A. (1992). Danish randomized trial comparing breast conservation therapy with mastectomy: six years of life-table analysis. Danish Breast Cancer Cooperative Group. *Journal of the National Cancer Institute. Monographs*.

[11] Voogd A. C., Nielsen M., Peterse J. L. (2001). Differences in risk factors for local and distant recurrence after breast-conserving therapy or mastectomy for stage I and II breast cancer: pooled results of two large European randomized trials. *Journal of Clinical Oncology*.

[12] Botteri E., Bagnardi V., Rotmensz N. (2010). Analysis of local and regional recurrences in breast cancer after conservative surgery. *Annals of Oncology*.

[13] Lapach S. N., Chubenko A. V., Babich P. N. (2002). *Statistics in Science and Business*.

[14] Fitzgibbons P. L., Page D. L., Weaver D. (2000). Prognostic factors in breast cancer. College of American Pathologists Consensus Statement 1999. *Archives of Pathology & Laboratory Medicine*.

[15] Huang E., Buchholz T. A., Meric F. (2002). Classifying local disease recurrences after breast conservation therapy based on location and histology: new primary tumors have more favorable outcomes than true local disease recurrences. *Cancer*.

